# Occurrence of *Salmonella* Typhimurium and its monophasic variant (4, [5],12:i:-) in healthy and clinically ill pigs in northern Italy

**DOI:** 10.1186/s40813-021-00214-1

**Published:** 2021-04-26

**Authors:** Mario D’Incau, Cristian Salogni, Stefano Giovannini, Jessica Ruggeri, Federico Scali, Matteo Tonni, Nicoletta Formenti, Flavia Guarneri, Paolo Pasquali, Giovanni Loris Alborali

**Affiliations:** 1 Istituto Zooprofilattico Sperimentale della Lombardia e dell’Emilia-Romagna “Bruno Ubertini”, via Bianchi 9, 25124 Brescia, Italy; 2 Department of Food Safety, Nutrition and Veterinary Public Health, Istituto Superiore di Sanità, viale Regina Elena 299, 00161 Roma, Italy

**Keywords:** *Salmonella* Typhimurium, Pig, Epidemiology

## Abstract

**Background:**

The serovar Typhimurium (4, [5],12:i:1,2), is the most frequently isolated serovar in case of salmonellosis in pigs in Europe and its monophasic variant (4, [5],12:i:-) has been increasingly responsible for *Salmonella* outbreaks in humans. A total of 25,215 samples were collected, during the years 2002–2017, from 1359 pig farms located in Northern Italy. Samples were collected from different material sources including fecal samples, rectal swabs, gut content and different organs.

**Results:**

*Salmonella* was isolated in 15.80% of samples and, among the isolates, 733 were typed as *Salmonella* Typhimurium (ST) or its monophasic variant (MST). Over time, there was an increase of isolation of MST which outnumbered ST. Most of the strains were isolated in animals during the weaning stage and the growing – fattening period whereas the clinical cases were mainly present in young pigs after weaning.

**Conclusions:**

This study confirms the presence of ST and MST in pig farms although, considering the total of isolated serotypes, with lower percentages than previously reported.

In the last few years, ST has increasingly been replaced by MST suggesting that MST has a competitive advantage over ST, probably due to its different antigenicity and pathogenicity which renders the infection stealthier to recognize and control.

## Background

Pigs can be infected with a broad range of *Salmonella* serotypes some of which can cause clinical disease and, frequently, can contaminate meat products [1].

Apart from the serovar Choleraesuis of *S. enterica subsp. enterica*, a host-adapted serovar usually isolated in cases of septicemia, the serovar Typhimurium, is now the most frequently isolated serovar in case of illness in pigs in Europe [2–4] and in the United States [5].

Clinically ill pigs can develop, in the most severe cases, enterocolitis and exhibit diarrhea and dehydration. The disease most commonly develops in pigs with concurrent debilitating diseases, in conditions of poor hygiene that allow exposure to high doses of the organism, or in immunologically naive pigs. Mortality is variable. Most pigs have a complete recovery and eliminate the organism, but others may remain carriers and intermittent shedders for several months [1].

The monophasic variant of *Salmonella* Typhimurium (4, [5],12:i:-) has been increasingly responsible for *Salmonella* outbreaks in humans, being the third (after the serovars *Salmonella* Enteritidis and *Salmonella* Typhimurium) most commonly reported serovar in the EU in 2012 [6], and frequently reported across the world [7, 8]. This serovar, strongly associated with swine food chain, especially in Europe [9], was rarely identified before the mid-1990s but its isolation in both animals and humans, has increased in the last 20 years [4, 10–12].

*Salmonella* prevalence varies widely among farms and at different growth stages within the same farm and, due to the high number of factors and the complex relationships among pathogen and host, definitive understanding of the transmission, shedding and carrier states of salmonellae are still difficult [1].

The aim of this study is to describe and evaluate the occurrence, over a 15-year period, of *Salmonella* Typhimurium (ST) and its monophasic variant *Salmonella enterica subsp. enterica* 4, [5],12:i:- (MST) in pigs and its association with clinical conditions.

## Materials and methods

### Strains isolation

A total of 25,215 samples were collected, during the years 2002–2017, from 1359 pig farms located in Northern Italy where clinical enteric forms or cases of on-farm mortality occurred. In particular, in each farm around 20 samples per year were collected either from living animals (fecal samples or rectal swabs) or from carcasses (gut content and different organs like spleen, liver and lymph nodes collected at necropsy) and sent to our lab by farm vets.

The isolation and identification of *Salmonella* isolates were carried out always by the same lab, initially in accordance with ISO 6579:2002 and later, for samples collected since 2007, in accordance with ISO 6579:2007 amendment 1. Briefly, the samples were pre-enriched with Buffered Peptone Water (BPW) and incubated at 37 °C ± 1 °C for 18 ± 2 h. The samples were then transferred, for enrichment, to Rappaport – Vassiliadis Soya Broth (RVS), incubated at 41.5 °C ± 1 °C for 24 ± 3 h, and Mueller – Kauffmann Tetrathionate with Novobiocin Broth (MKTTn), incubated at 37 °C ± 1 °C for 24 ± 3 h (for 2002–2007 samples: ISO 6579:2007), and (for 2007–2017 samples: ISO 6579:2007 amendment 1) to a Modified Semisolid Rappaport – Vassiliadis (MSRV) agar medium incubated at 41.5 °C ± 1 °C for 24 ± 3 h. MSRV agar plates were incubated for further 24 ± 3 h if negative. Enrichment cultures were used to inoculate two solid media incubated at 37 °C ± 1 °C for 24 ± 3 h: Xylose Lysine Deoxycholate agar (XLD) and Brilliant Green Agar (BGA).

Colonies of presumptive *Salmonella* were sub-cultured on Triple Sugar Iron (TSI) agar at 37 °C ± 1 °C for 18 ± 2 h and further, in accordance with ISO 6579:2007, identified biochemically and confirmed as *Salmonella* by slide agglutination using a polyvalent O antiserum.

### Strain serotyping

The complete serological characterization of *Salmonella* was performed by slide agglutination for the determination of somatic antigens, while, for the determination of flagellar antigens, the method of tube agglutination was followed according to the Spicer [13] technique, modified by Morris et al. [14].

In particular, the characterization of monophasic variant of *Salmonella* Typhimurium, was performed through two consecutive phase inversions by passaging through a U-shaped glass tube containing semisolid agar with H:i antiserum. The isolates that still did not display the second phase after the first and the second passage were considered, phenotypically, monophasic.

### Phage-typing

Phage-typing was performed at the Italian National Reference Centre for Animal Salmonellosis according to Anderson et al. [15]. Typing of MST strains started in 2011 and for this reason only 225 isolates were typed. The total number of typed ST strains was 235.

### Clinical case definition

We considered a “clinical case” (C) as an illness of variable severity manifested by enteric signs with presence of *Salmonella* and in absence of isolation of other enteric pathogens. When *Salmonella* isolation occurred and no enteric signs were shown the condition was referred as a “non-clinical case” (NC).

### Statistical methods

Statistical analysis was performed using GraphPad 6.0 for MAC OS X (GraphPad Software Inc.; San Diego; CA) and SAS (SAS Institute, Inc., Cary, North Carolina) softwares. Differences in proportions were estimated using Fisher’s exact test. Differences in ST and MST prevalence among years were evaluated through Chi-squared test. A *P* value less than 0.05 was considered statistically significant while a value between 0.05 and 0.1 will be defined as trend.

## Results

### Serotyping results

**Fig. 1:**
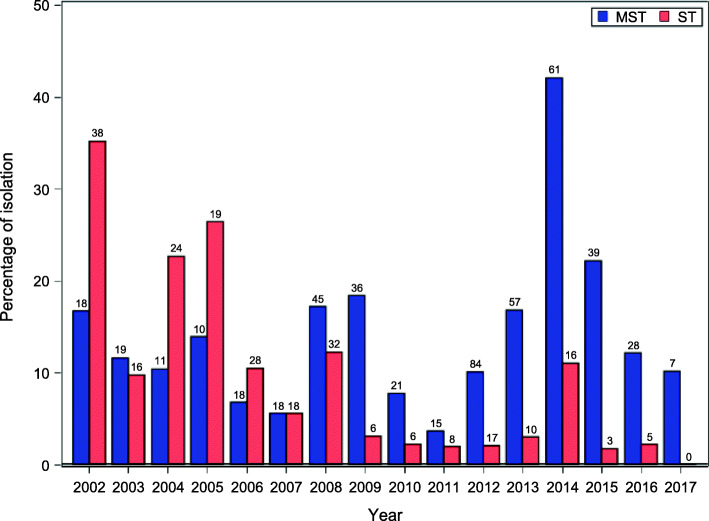
Distribution of the isolates (percentages on the total of positive samples) of *Salmonella* Typhimurium (ST) and its monophasic variant (MST), during the period of the study (2002–2017)

*Salmonella* was isolated from 3983 out of 25,215 fecal samples (15.80%). Among the isolates, 246 were typed as *Salmonella* Typhimurium and 487 as its monophasic variant. Two hundred eighty-five out of 1359 farms (20.97% of the total) resulted positive for the serovars investigated in this study. The distribution of the isolates, during the considered period, is showed in Table 1 and Fig. 1. Overall, it is possible to see two distinct phases. The number of ST isolates were slightly greater than that of MST isolates from 2002 to 2007. Thereafter, since 2008, it is possible to see a steady increase of the presence of MST over ST. Prevalence of both ST (χ^2^_15_ = 363.3019, *p* < 0.0001) and MST (χ^2^_15_ = 217.5584, *p* < 0.0001) varied significantly depending on the study year.

**Table 1:** Number of MST and ST isolates and related percentages on the total of positive samples

Year	Total positive samples	ST		MST		ST + MST	
		n	%	n	%	n	%
2002	108	38	35.19	18	16.67	56	51.85
2003	164	16	9.76	19	11.59	35	21.34
2004	106	24	22.64	11	10.38	35	33.02
2005	72	19	26.39	10	13.89	29	40.28
2006	268	28	10.45	18	6.72	46	17.16
2007	325	18	5.54	18	5.54	36	11.08
2008	262	32	12.21	45	17.18	77	29.39
2009	196	6	3.06	36	18.37	42	21.43
2010	272	6	2.21	21	7.72	27	9.93
2011	416	8	1.92	15	3.61	23	5.53
2012	834	17	2.04	84	10.07	101	12.11
2013	339	10	2.95	57	16.81	67	19.76
2014	145	16	11.03	61	42.07	77	53.10
2015	176	3	1.70	39	22.16	42	23.86
2016	231	5	2.16	28	12.12	33	14.29
2017	69	0	0.00	7	10.14	7	10.14
**Total**	**3983**	**246**	**6.18**	**487**	**12.23**	**733**	**18.40**

**Fig. 2:**
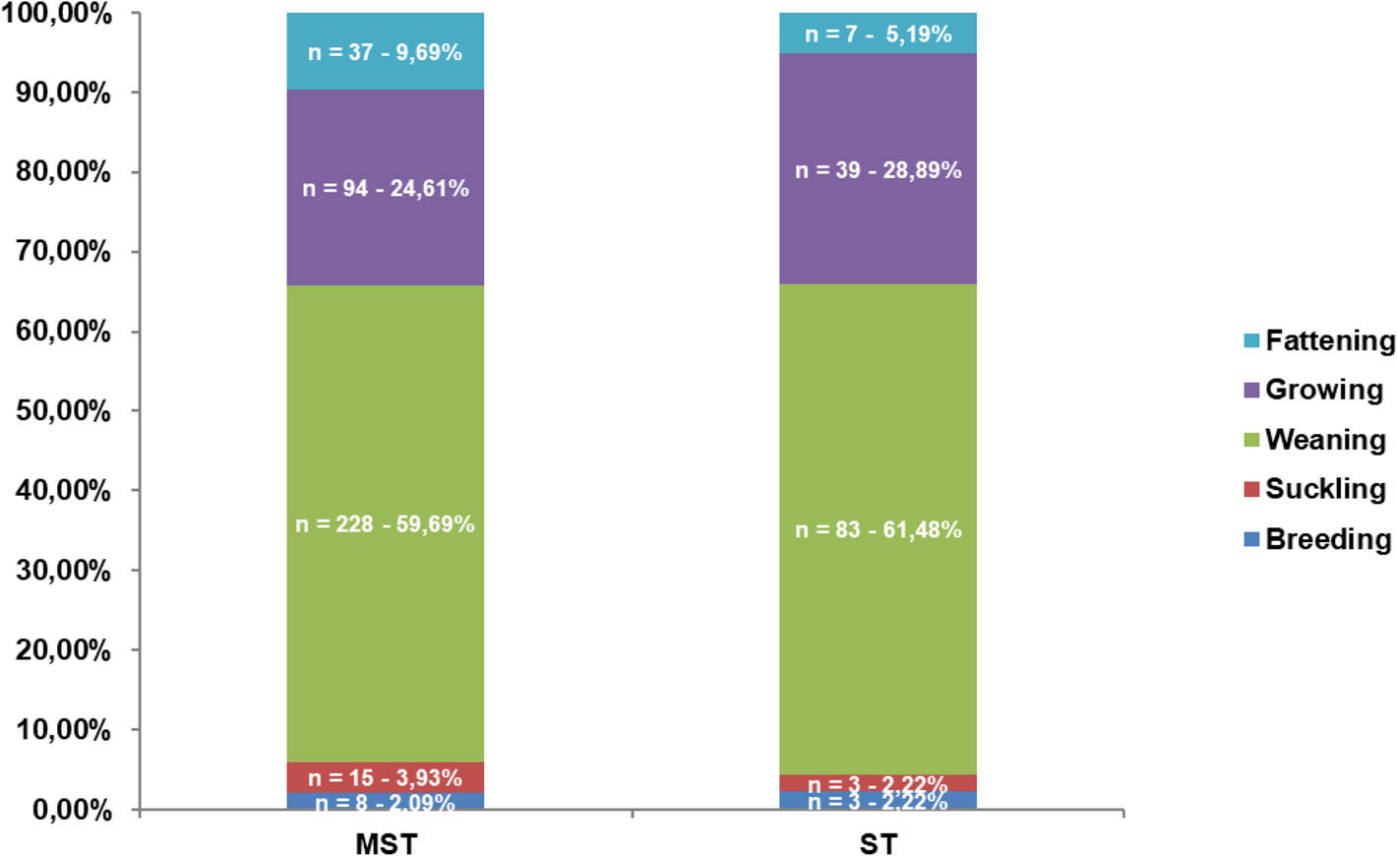
Distribution of the isolates of *Salmonella* Typhimurium (ST) and its monophasic variant (MST) related to the production stages

Five hundred seventeen out of 733 collected isolates came from pigs whose different ages were known: amongst them, most of the MST and ST strains were isolated in animals during the weaning (since 30 days of age till 25/30 kg weigh) stage (*n* = 311; 60.15%) and the growing (25/30 kg – 60 kg weigh) – fattening (60 kg weigh to slaughtering) period (*n* = 177; 34.24%) whereas the number of isolates from breeders (*n* = 11; 2.13%) as well as from suckling piglets (*n* = 18; 3.48%) was low. Similar patterns of distribution were observed considering ST and MST separately (Fig. 2).

Clinical signs were associated with 114 out of 246 (46.34%) isolates of ST and 184 out of 487 (37.78%) isolates of MST (Table 2). Although it seems that the association of MST to clinical signs is lower than in ST, the difference only approached the statistical significance (*P* = 0.08).

**Table 2:** Association between clinical conditions and the presence of *Salmonella* Typhimurium (ST) and its monophasic variant (MST)

	Clinical signs present (number of isolates)	Clinical signs absent (number of isolates)	Total number of isolates
**ST**	114	132	246
**MST**	184	303	487
**Total**	**298**	**435**	**733**

**Fig. 3:**
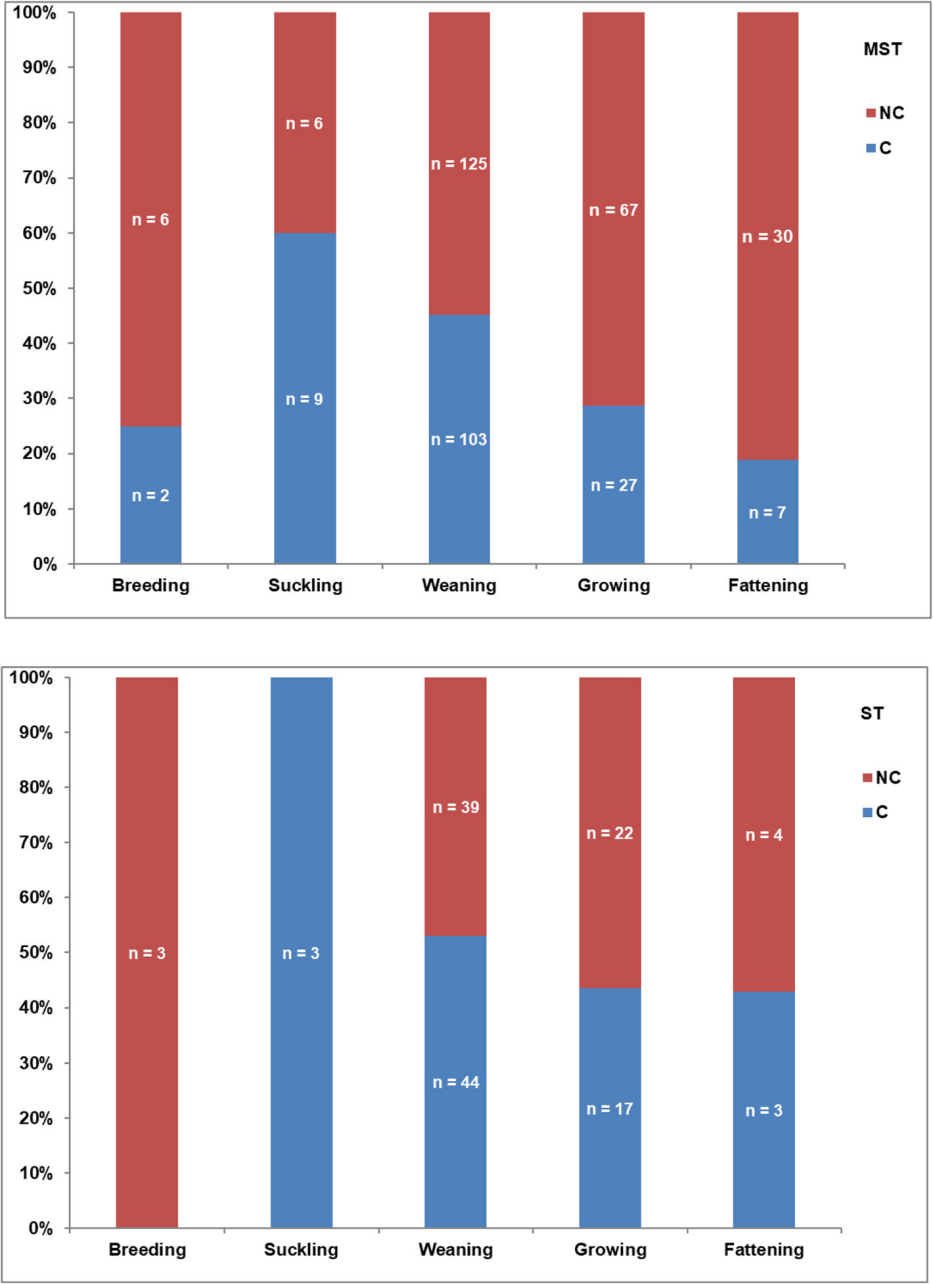
Percentage of the isolates of *Salmonella* Typhimurium (ST) and its monophasic variant (MST) associated (C) or not associated (NC) with clinical illness related to the production stages

Figure 3 shows the percentage of strains (on the total of MST and ST strains) associated with clinical illness referred to the single production stage. Most of the clinical cases were present in young pigs after weaning, while in fatteners and breeders the occurrence of clinical signs were lower. When infected with MST, clinical signs were present in 45.17% (103/228) and 25.95% (34/131) of pigs, in weaners and growers-fatteners respectively, while in case of isolation of ST, clinical signs were present in 53.01% (44/83) and 43.48% (20/46) of pigs, respectively.

### Phage typing results

The isolates belonged to 23 different phage types (Table 3), and eight of them were common to both serovars. The most frequently isolated phage types were DT193 (62 isolates) and DT120 (47 isolates), followed by U311 (45 isolates) and U302 (29 isolates). Other types found were DT104 (18 isolates), DT20A (16 isolates) and DT208 (11 isolates). DT193 was the most frequent type among MST strains while U302 was the most common among ST strains. Forty-nine isolates showed a pattern which did not conform (RDNC) to any defined pattern, and 149 could not be phage typed (NT).

**Table 3:** Phage-types associated with isolates of *Salmonella* Typhimurium (ST) and its monophasic variant (MST) and related percentages on the total of each phage-type

Phage type	MST		ST		Total phage-typed
	n	%	n	%	
DT193	50	80.65	12	19.35	62
DT120	36	76.60	11	23.40	47
U311	28	62.22	17	37.78	45
U302	4	13.79	25	86.21	29
DT104		0	18	100.00	18
DT20A	13	81.25	3	18.75	16
DT208		0	11	100.00	11
DT110	1	14.29	6	85.71	7
DT104B		0	5	100.00	5
DT12		0	5	100.00	5
DT7VAR		0	3	100.00	3
DT1	1	50.00	1	50.00	2
DT32	1/	50.00	1	50.00	2
DT138		0	1	100.00	1
DT193A	1	100.00		0	1
DT194		0	1	100.00	1
DT195		0	1	100.00	1
DT27		0	1	100.00	1
DT36		0	1	100.00	1
DT7		0	1	100.00	1
DT7A	1	100.00		0	1
DT99		0	1	100.00	1
U310		0	1	100.00	1

## Discussion

The results presented here were obtained from farms, located in Northern Italy in a high-density pig population area, and checked when clinical enteric forms or cases of on-farm mortality occurred. We focused on ST and MST, considering the prominent role of these two serovars in the pig population [4, 12].

ST and MST represented 12.23% and 6.18, respectively of the *Salmonella* serovars isolated, low percentages compared to those from other reports. Indeed, recent studies suggested that ST and MST represent between 40 and 50% of the Italian isolates, with MST increasing from 9.66 to 46.34% in the last 10 years [16, 17]. A similar increasing has also been reported in other countries [12]. Although it may seem that, in this study, a lower prevalence than those available in the literature to date were recorded, a comparison can not actually be made because in this study we used a clinical sampling with the presence of the enteric clinical form in the herd as inclusion criterion. Therefore, the fact that our sampling was not randomly performed, but following clinical criteria, does not make possible a comparison with other studies. The distribution of the studied serovars, during the considered period, highlighted a predominance of ST on MST in the first period and, since 2008, a reversion of this tendency with MST becoming more predominant over the following years. Indeed after 2009, MST subsequent recorded peaks were likely related to cyclic outbreaks and to the hypothesized pigs’ role of reservoir for this serovar [18].

The data reported here are in accordance with recent reports where the increasing prevalence of MST is well documented, in particular in the United Kingdom, Poland and Malta [6, 19, 20]. In the United Kingdom, MST represented 60.7% of the *Salmonella* isolates obtained from a surveillance program in pigs in 2015 [21].

It is conceivable to hypothesize that MST has a selective advantage over ST. It was suggested that a number of factors (i.e. involvement of prophages and antigenic changes) can cause a reduced immune response to MST in herds when compared to ST [4]. More recently, a comparative whole-genome sequencing and phylogenomic analysis of MST isolates from the United Kingdom and Italy during the period 2005–2012, revealed a high level of microevolution that may affect antigenicity, pathogenicity, and transmission [22].

Although the increase of the number of the MST isolates coincides with the revision of the isolation method, in order to rule out any influence of the testing method upon the results, it is important to highlight that the analyses were performed according to ISO methods: the change of the enrichment media can not affect the suitability of detecting any of the known motile Salmonella serovars [23]. The replacements of two enrichment broth media with a semi-solid medium leads, on the other hand, to a better selection on the background flora able to grow in the broths and potentially able to mask the isolation of *Salmonella* strains [23].

When considering the production stage, both ST and MST showed their highest presence in the weaning and growing period as reported previously [24, 25]. A comparison between the prevalence of ST and MST in different production stages showed no significant differences. Overall, these findings suggest a higher level of susceptibility in younger pigs, irrespective of the serovars involved. Pigs can become infected at any production stage but the decline of maternal antibodies after weaning makes younger pigs more susceptible to the infection [26].

When considering the association between clinical signs and isolates, we observed that clinical signs were associated more to ST than to MST and that most of clinical cases were present in young pigs after weaning. These data, although only approaching significance, are supportive of a competitive advantage of MST over ST.

The phage-typing highlighted the prevalence of four types representing about 70% of the typed isolates (DT193, DT120, U311, U302) and this has been a common feature of European isolates for the last 20 years [11, 27, 28]. DT193 has to be regarded as an important phage type also for ST, considering its increase in Europe in the last years [29] and its role in human cases of salmonellosis.

## Conclusions

In conclusion, this study confirms the presence of ST and MST in pig farms although, considering the total of isolated serotypes, with lower percentages than previously reported.

In the last few years, ST has increasingly been replaced by MST suggesting that MST has a competitive advantage over ST, probably due to its different antigenicity and pathogeneicity which renders the infection stealthier to recognize and control. More detailed studies should be undertaken to assess the mechanisms underpinning the competitive advantage of MST over ST in pigs.

## Data Availability

The datasets used and/or analysed during the current study are available from the corresponding author on reasonable request. The dataset supporting the conclusions of this article is included within the article.

## Abbreviations

ST: Salmonella Typhimurium
MST: Salmonella Typhimurium monophasic variant
EU: European Union
RDNC: Result does not conform
NT: Not typed
ISO: International Standard Organization
BPW: Buffered Peptone Water
RVS: Rappaport – Vassiliadis Soya
MKTTn: Mueller – Kauffmann Tetrathionate with Novobiocin
MSRV: Modified Semisolid Rappaport – Vassiliadis
XLD: Xylose Lysine Deoxycholate
BGA: Brilliant Green Agar
TSI: Triple Sugar Iron
C: Clinical case
NC: Non-clinical case
